# Adult T-cell leukemia/lymphoma in a Caucasian patient after sexual transmission of HTLV-1

**DOI:** 10.1186/1742-4690-12-S1-P76

**Published:** 2015-08-28

**Authors:** David Sibon, Olivier Cassar, Isabelle Duga, Chantal Brouzes, David Ghez, Christophe Pasquier, Claire Sibon, Alexandra Desrames, Franck Mortreux, Eric Wattel, Ali Bazarbachi, Antoine Gessain, Olivier Hermine

**Affiliations:** 1 Hématologie Adulte, Hôpital Universitaire Necker–Enfants Malades, Assistance Publique– Hôpitaux de Paris (APHP), Paris, France; 2 INSERM U1163 and CNRS ERL 8254 Bases Cellulaires et Moléculaires des Désordres Hématologiques, France; 3 Institut Imagine, Université Paris Descartes– Sorbonne Paris Cité, Paris, France; 4 Institut Pasteur, Unité d'Epidémiologie et Physiopathologie des Virus Oncogènes, Département de Virologie, Paris, France; 5 CNRS, UMR 3569, Rue du Docteur Roux, F-75015 Paris, France; 6 Département de Pathologie, Hôpital Purpan, Toulouse, France; 7 Laboratoire d'Hématologie, Hôpital Universitaire Necker–Enfants Malades, Assistance Publique–Hôpitaux de Paris (APHP), Paris, France; 8 Hématologie, Institut Gustave-Roussy, Villejuif, France; 9 Laboratoire de Virologie, Hôpital Purpan, Toulouse, France; 10 Département de Dermatologie, Hôpital Ambroise-Paré, Boulogne-Billancourt, France; 11 Laboratoire d'Oncovirologie et Biothérapies, CNRS UMR 5239, Centre Hospitalier Lyon Sud, Lyon, France; 12 Department of Internal Medicine, American University of Beirut, Beirut, Lebanon

## 

Adult T-cell leukemia/lymphoma (ATLL) is an aggressive T-cell lymphoproliferation caused by human T-cell lymphotropic virus type-1 (HTLV-1). This oncogenic human retrovirus can be acquired by mother-to-child transmission through prolonged breast-feeding, sexual transmission, or from transfused infected blood cells or intravenous drug abuse. HTLV-1 infects approximately 5–10 million individuals worldwide and, among them, 1–5% will develop ATLL during their lifetime. Four major geographic molecular subtypes (genotypes) have been reported including the cosmopolitan a-subtype, the Central African b-subtype, the Central African/Pygmies d-subtype and the Australo-Melanesian c-subtype. The results of several studies showed that most cases of ATLL develop in individuals who have been infected with HTLV-1 as young children via their mothers’ breast milk. The very rare ATLL cases observed following transfusion or sexual transmission are still being debated. Here, we report on a Caucasian French patient, with HTLV-1–seronegative parents, who developed ATLL, characterized by a clonal T cell skin proliferation of CD4+ and CD25+ cells, 18 years after highly probable sexual transmission of HTLV-1 through repeated unprotected sexual intercourse with a Cameroonian woman. Indeed, genotyping of the patient's virus revealed infection with an HTLV-1 b-subtype strain, typically of Central African origin, especially Cameroon. This case definitively confirms the hypothesis that ATLL can develop, albeit rarely, after infection during adulthood, outside breast-feeding.

